# Tissue-Specific B-Cell Dysfunction and Generalized Memory B-Cell Loss during Acute SIV Infection

**DOI:** 10.1371/journal.pone.0005966

**Published:** 2009-06-19

**Authors:** Sandrine Peruchon, Nada Chaoul, Chantal Burelout, Benoit Delache, Patricia Brochard, Pascale Laurent, Fabrice Cognasse, Sophie Prévot, Olivier Garraud, Roger Le Grand, Yolande Richard

**Affiliations:** 1 Atomic Energy Commission, Institute of Emerging Diseases and Innovative Therapies, Division of Immuno-Virology, UMR-E1, Univ. Paris-Sud, Orsay, France; 2 Groupe sur l'Immunité des Muqueuses et Agents Pathogènes (GIMAP), Faculté de Médecine and Etablissement Français du Sang (EFS) Auvergne-Loire, Saint Etienne, France; 3 Service d'Anatomie et Cytologie pathologiques, AP-HP, Hôpital A. Béclère, Clamart, France; Karolinska Institutet, Institution for Laboratory Medicine, Sweden

## Abstract

**Background:**

Primary HIV-infected patients display severe and irreversible damage to different blood B-cell subsets which is not restored by highly efficient anti-retroviral therapy (HAART). Because longitudinal investigations of primary HIV-infection is limited by the availability of lymphoid organs, we studied the tissue-specific B-cell dysfunctions in acutely simian immunodeficiency virus (SIV) mac251-infected Cynomolgus macaques.

**Methods and Findings:**

Experiments were performed on three groups of macaques infected for 14, 21 or 28 days and on three groups of animals treated with HAART for two-weeks either initiated at 4 h, 7 or 14 days post-infection (p.i.). We have simultaneously compared changes in B-cell phenotypes and functions and tissue organization of B-cell areas in various lymphoid organs. We showed that SIV induced a steady decline in SIgG-expressing memory (SIgD^−^CD27^+^) B-cells in spleen and lymph nodes during the first 4 weeks of infection, concomitant to selective homing/sequestration of B-cells to the small intestine and spleen. SIV non-specific Ig production was transiently increased before D14p.i., whereas SIV-specific Ig production was only detectable after D14p.i., coinciding with the presence of CD8^+^ T-cells and IgG-expressing plasma cells within germinal centres. Transient B-cell apoptosis on D14p.i. and commitment to terminal differentiation contributed to memory B-cell loss. HAART abrogated B-cell apoptosis, homing to the small intestine and SIV-specific Ig production but had minimal effect on early Ig production, increased B-cell proportions in spleen and loss of memory B-cells. Therefore, virus–B-cell interactions and SIV-induced inflammatory cytokines may differently contribute to early B-cell dysfunction and impaired SIV/HIV-specific antibody response.

**Conclusions:**

These data establish tissue-specific impairments in B-cell trafficking and functions and a generalized and steady memory B-cell loss in secondary lymphoid organs. Characterization of underlying mechanisms would be helpful in designing new therapeutic strategies to dampen B-cell activation and increases HIV/SIV specific antibody response.

## Introduction

B-cell dysfunction represents a central feature of HIV infection and an important pathogenic mechanism [1]–[5]. In the absence of highly active antiretroviral therapy (HAART), HIV-1 infection is associated with a wide range of B-cell defects, including polyclonal hypergammaglobulinemia and the presence of immature/transitional CD10+ or exhausted CD27 negative B-cells in blood [5], [6]. Decreased expression of CXCR5 on blood B-cells [7] but increased proportions of CD38-expressing B-cells have been described as a consequence of abnormal trafficking of germinal centre (GC)-like B-cells into blood [8]. More recently, Cagigi et al have shown that the decrease in CXCR5 expression is concomitant to abnormal CXCL13 production by peripheral and lymph node B-cells and increased B-cell responsiveness to CXCL13 in HIV-1 infected patients with low CD4+ T-cells counts [9]. As CXCR5/CXCL13 pair is essential for the entry of naive B-cells and marginal zone (MGZ) B-cells into follicles [10], [11], altered expression of this chemokine receptor-ligand pair may contribute to abnormal B-cell trafficking during the course of HIV-1 infection. In secondary lymphoid tissue from HIV-1 infected individuals, follicular hyperplasia and alterations in the architecture of GC and splenic MGZ were observed [12]–[14]. Despite polyclonal activation, the humoral response is strongly impaired, resulting in a decreased response to natural or vaccine T-independent and T-dependent antigens [15] and a loss of peripheral memory B-cells [16]–[18]. Most of these defects are considered as the hallmarks of the chronic phase of infection and are frequently correlated with increased plasma viral load (pVL) and loss of CD4+ cells [5]. However, recent studies in humans [15], [19] suggest that early activation has a major role in shaping B-cell (memory and plasma cell) repertoire and trafficking [19]–[21]. In particular, primary HIV-infected patients have severe and irreversible damage to several blood B-cell subsets, which is not counteracted by HAART despite an increase in CD4+ T-cell counts and a decreased viral load [3].

The study of primary HIV-1 infection in humans is limited by the availability of lymphoid organs and the difficulties associated with performing longitudinal investigations using tissues other than blood or tonsils. We thus used a model of experimental pathogenic infection in cynomolgus macaques—a well-known, suitable model that reproduces long-lasting HIV-1 disease [22]—to investigate B-cell dysfunctions during acute HIV infection. Experiments were performed on three groups of macaques infected by SIV_mac251_ strain for 14, 21 or 28 days (placebo-treated groups) and on three groups of animals treated with HAART for two-weeks either initiated at 4 h, 7 or 14 days post-infection (p.i.) and sacrificed at D14, 21 and D28 p.i. (HAART-treated groups). After having characterized naive, memory and MGZ B-cell subsets in various lymphoid organs from non-infected animals, we have compared the changes in these B-cell subsets in blood, lymph nodes (LN) and spleen of placebo- and HAART-treated macaques and examined whether correlations could be established with immunological or virological parameters. Changes in B-cell functions *in vitro* (proliferation, apoptosis or immunoglogulins (Ig) production) as well as in tissue organization of areas populated by B-cells within lymphoid tissues (spleen, small intestine and mesenteric LN) were concomitantly examined in placebo- or HAART-treated animals.

Our data show that SIV induced a transient increase in B-cell apoptosis and SIV non-specific Ig production by D14p.i., a steady loss of memory B-cells in spleen and peripheral LN but promoted preferential B-cell trafficking to the small intestine and spleen. HAART initiated 4 hp.i. strongly decreased B-cell apoptosis and B-cell seeding of gut mucosa, but not memory B-cell loss. The production of SIV non-specific IgG was similar in placebo- and HAART-treated animals whereas that of SIV-specific antibodies, only detectable after D14p.i., was abrogated by HAART initiated on D7p.i.. These data establish tissue-specific impairments in B-cell trafficking and functions but a generalized and steady memory B-cell loss in secondary lymphoid organs. Our results provide a clearer understanding of the effects of SIV/HIV-1 on the B-cell compartment during the acute/primary phase of infection.

## Results

### SIV infection induces a B-cell increase in spleen

We compared the proportions of lymphocyte subsets in blood, spleen and peripheral (inguinal and axillary) LN from various groups of animals infected for 14, 21 or 28 days. Baseline values for blood samples were obtained from each animal before infection, whereas those for spleen and LN were obtained from non-infected animals (controls).

**Figure 1:**
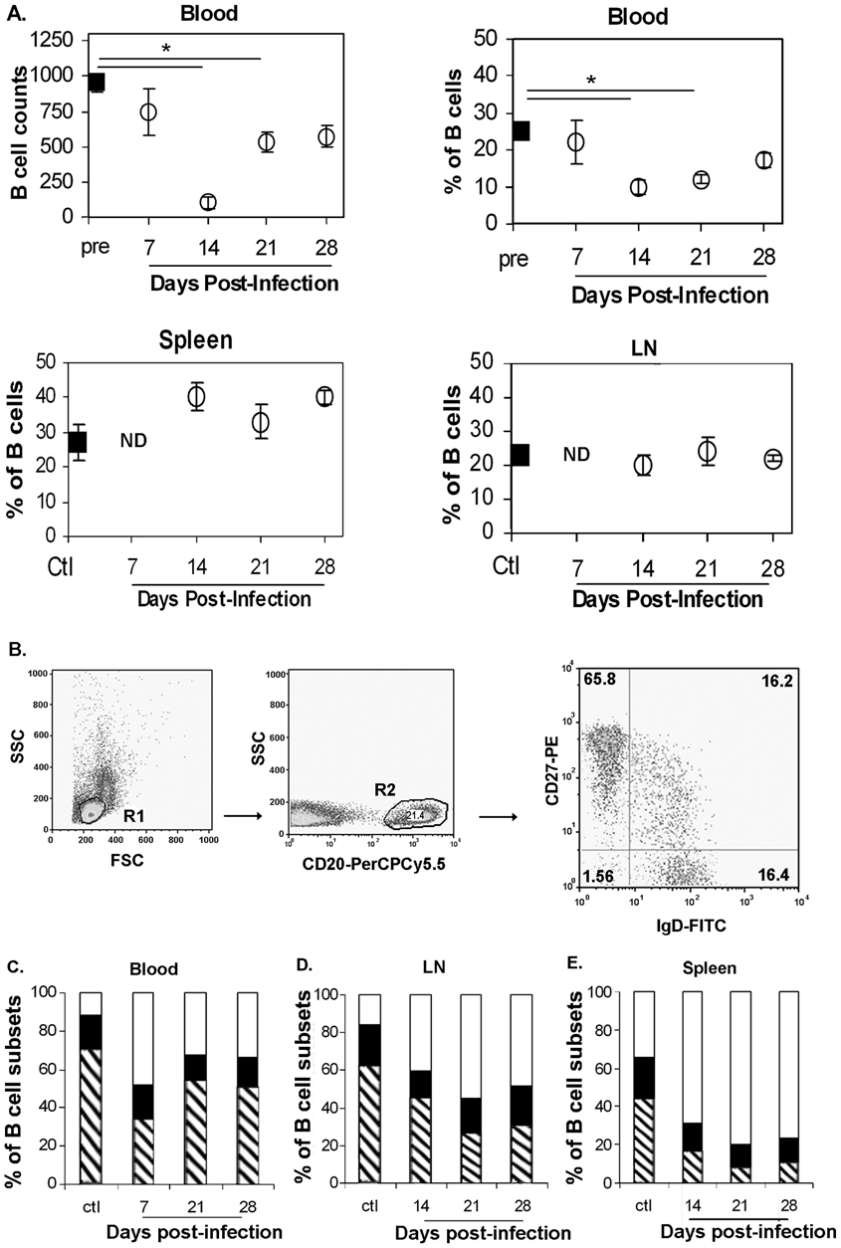
Changes in B-cell subsets in blood, peripheral LN and spleen of SIV-infected animals. (A) Cell suspensions from various organs were analyzed by flow cytometry for CD20 expression. Absolute cell numbers (cells/µl) or percentages of blood B-cells before infection (pre, *black square*, n = 13), on D7, 14, 21 p.i. (*open circle*, n = 5 per group) or on D28p.i. (*open circle*, n = 3). *, *p* values<0.05 are shown. ND: Not Done. (B) CD27 and SIgD expression was studied by 3-paramater immunofluorescence analysis on CD20-gated populations from various organs. Cells are gated on forward and side scatter (R1), then on CD20 (R2). Expression of CD27 and SIgD in CD20+ cells is shown on a representative dot plot of PBMC staining. The percentage of positive cells is indicated in each quadrant. Flow cytometry plots depict Log10 Fluorescence. (C–E) The mean percentages of naive (SIgD+ CD27−, *open area*), memory (SIgD− CD27+, *hatched area*) and MGZ (SIgD+ CD27+, *black area*) B-cells for each group of animals are shown in panels C to E. Data in blood before (n = 3) or after exposure to SIV (n = 3 per group) are shown in panel C. Data from spleen of non-infected or SIV-infected animals (n = 3 for all groups) are shown in panel D. Data in LN from non-infected animals (Ctl, n = 4) or SIV-infected animals (n = 5 for all groups) are shown in panel E.

Consistent with previous reports [23], [24], SIVmac251 infection induced a decrease in blood CD4^+^ and CD8^+^ T-cell and NK-cell counts, detectable by D7 p.i., but reaching their nadir on D14 p.i., with reductions of 53% for CD4^+^, 49% for CD8+ and 85% for NK cells (**Table 1** **)**. Plasma VL was inversely correlated with CD4^+^ (*Rho* = −0.57, *p* = 0.05) and CD8+ (*Rho* = −0.65, *p* = 0.03) T-cell counts (**supplemental** **Figure S1**). In these SIV-infected animals, the number and percentage of B-cells (**Figure 1.A**) decreased to a nadir reached on D14 p.i. (87% and 60% decrease, respectively) and remaining at 35% and 40% below the baseline values on D28 p.i.. B-cell counts correlated with percentages of B-cells (*Rho* = 0.57, *p* = 0.02) and CD4^+^ cell number (*Rho* = 0.88, *p*<0.01) and inversely correlated with pVL *(Rho* = −0.79; *p*<0.01) in SIV-infected animals (**supplemental** **Figure S1**). In contrast to blood, the proportion of B-cells in the spleen steadily increased, with a 40% increase detected from D14 p.i. on (40±4%, 33±5% and 40±2% on D14, 21 and 28p.i., respectively versus 27±5% in non-infected animals) and the proportion of B-cells varied by less than 10% of baseline values in LN (20±3%, 24±6% and 22±1% on D14, 21 and 28p.i., respectively versus 23±2% in non-infected animals) (**Figure 1.A**). These data suggest that SIV infection induces a preferential accumulation of B-cells in the spleen during the acute phase of infection.

**Table 1:** Peripheral B cells (CD20+) strongly decrease during infection by SIVmac251.

Days after infection	SIV	pVL^a^	CD4+CD3+				CD8+CD3+				NK^b^				CD20			
		copies/ml	number/µl			% modulation	number/µl			% modulation	number/µl			% modulation	number/µl			% modulation
0	−	−	1493	±	167		1464	±	310		911	±	186		992	±	104	
7	+	(7±3)×10^4^	1173	±	219^c^	(−)13±9	1108	±	334^c^	(−)16±11	536	±	180^c^	(−)29±14	748	±	167	(−)22±12
0	−	−	573	±	66		770	±	74		988	±	322		918	±	154	
14	+	(20±10)×10^6^	256	±	103^c^	(−)53±16	386	±	149	(−)49±19	168	±	85^c^	(−)85±7	102	±	43^c^	(−)87±5
0	−	−	1493	±	167		1464	±	310		911	±	86		992	±	104	
21	+	(31±5)×10^5^	1181	±	122	(−)5±13	1651	±	177	(+)44±17	666	±	105^c^	(−)1±18	531	±	66^c^	(−)42±6
0	−	−	1134	±	215		1028	±	205		351	±	28		961	±	206	
28	+	(47±13)×10^4^	877	±	81	(−)16±13	1541	±	424	(+)60±46	521	±	209	(+)40±46	568	±	77	(−)35±9

a Plasma Viral Load.

b NK subset defined as CD8+CD3−.

c *p*<0.05 compared with animals before infection.

All values are means±SEM.

### Memory B-cell loss is induced early in acute SIV infection

Consistent with previous data obtained in humans [25]–[27], the combined analysis of CD20, CD27 and Surface IgD (SIgD) expression allows the delineation of three blood B-cell subsets in the macaque: SIgD^+^CD27− naive B-cells, SIgD−CD27^+^ memory B-cells and SIgD^+^CD27^+^ MGZ B-cells (**Figure 1.B**). In constrast to humans, we found that the proportion of peripheral memory B-cells was higher than that of naive B-cells (68±4% versus 11±2% of B-cells, respectively) in non-infected macaques (**Figure 1.C**). The proportions of blood MGZ B-cells were similar in humans (12±2%) [25] and macaques (17±5%). In LN, memory cells accounted for 59±5%, naive cells for 15±4% and MGZ B-cells for 22±6% of the total B-cell population (**Figure 1.D**), whereas they accounted for 39±10%, 30±5% and 17±5% of the total B-cell population in spleen (**Figure 1.E**). In agreement with our data, Vugmeyster et al reported that smaller proportions of naive (CD20^+^ CD27−) B-cells and higher proportions of CD20^+^CD27^+^ B-cells are present in blood and LN of cynomolgus macaques than those in humans [28]. A population of GC B-cells of variable size has been characterized in human lymphoid organs by the co-expression of CD20 antigen with CD10, CD38 and CD77 antigens. Unfortunately, staining with any of the specific antibodies (Ab) was not suitable to define a similar GC population in macaques (*data not shown*).

SIV infection induced an early but transient loss of peripheral memory B-cells, with a two- and 1.4-fold decrease observed in the percentage of cells at D7p.i. and D28p.i., respectively. A 4.3- and three-fold increase in peripheral naive B-cells was observed on D7 and D28p.i., respectively. Changes in MGZ B-cells were only detected on D21 and D28 p.i. (1.4- and 1.2-fold decrease, respectively) (**Figure 1.C**). In LN, the proportion of memory B-cells had decreased by factors of 1.3 by D14 p.i., 2.6 by D21p.i. and 2.2 by D28 p.i., respectively, whereas the percentage of naive B-cells increased by a factor of 2.7 from D14p.i. (**Figure 1.D**). The percentage of MGZ B-cells transiently decreased with a nadir observed at D14p.i. (1.6-fold decrease) and values approaching baseline at D21 p.i.. Results were similar for axillary and inguinal LN (*data not shown*). In spleen, memory B-cells were decreased by a factor of 2.3 by D14, 5.6 by D21 and 3.9 by D28 p.i.. The percentages observed for MGZ B-cells between D14 and D28 p.i. were between 1.2 and 1.5 times lower than baseline. In contrast, the percentage of naive B-cells steadily increased from D14p.i. by a factor of 2.3. (**Figure 1.E**). Differences in the proportion of memory and naive B-cells in blood, LN and spleen between non-infected and SIV-infected animals were significant (*p<0.05)* at each time point (**Figure 1C, D and E**). The percentage of MGZ B-cells only differed significantly between non-infected and SIV-infected animals in LN at D14p.i. (*p<0.05*). These results reveal a substantial depletion of memory and MGZ B-cells in all organs during the first four weeks of SIV infection. This depletion was transient in blood but longer lasting in lymphoid organs.

### Differential changes in memory B-cells from various lymphoid organs during SIV infection

It is well established that memory SIgD−CD27^+^ B-cells preferentially express SIgG/A over IgM in humans. We therefore quantified the relative levels of CD27^+^B-cells expressing SIgG and SIgM in macaques. The ratio between SIgM and SIgG in blood B-cells did not differ significantly between non-infected and SIV-infected animals at any time point tested during the four weeks of infection (**Table 2**). By contrast, this ratio progressively increased in LN and spleen, with 2.4- and 2.8-fold increases detected at D28 p.i. in these two tissues, respectively. These data suggest that switched memory B-cells were preferentially depleted in secondary lymphoid organs.

**Table 2:** Selective decreases in SIgG+, CD80+, CD86+ or CD95+ memory B cells.

Lymphoid organs	Days post-infection	n	Memory B cells												
			Ratio SIgM+/SIgG+^a^	% SIgD−CD27+			% SIgD− CD80+			% SIgD− CD86+			% SIgD− CD95+		
Blood	Ctl^b^	4	1.59±0.29	68	±	4	68	±	5	59	±	5	74	±	5
	7	3	1.61±0.09	34	±	3^c^	36	±	1^c^	31	±	3^c^	34	±	3
	21	3	2.15±0.65	53	±	4^c^	55	±	4	47	±	7	57	±	3
	28	3	1.59±0.09	50	±	10	49	±	11	41	±	11	51	±	10
LN	Ctl	4	0.60±0.07	59	±	5	30	±	8	34	±	4	62	±	10
	14	6	1.05±0.21	46	±	3	20	±	3	26	±	1	58	±	4
	21	5	1.13±0.20	23	±	6^c^	29	±	11	23	±	5	26	±	1
	28	6	1.88±0.24^c^	27	±	2^c^	27	±	1	22	±	2	33	±	2^c^
Spleen	Ctl	3	0.48±0.15	39	±	10	18	±	6	28	±	3	47	±	10
	14	3	1.15±0.12^c^	17	±	5	8	±	1	14	±	3^c^	ND		
	21	3	1.71±0.16^c^	7	±	1^c^	11	±	2	12	±	3^c^	ND		
	28	3	1.34±0.11^c^	10	±	1^c^	6	±	1	8	±	1^c^	16	±	1^c^

a The SIgM+ and SIgG+ populations are restricted to CD27+ B cell subsets.

b Ctl as control for non-infected animals.

c *p*<0.05 compared with non-infected animals.

All values are means±SEM.

As in humans, macaque memory B-cells can express CD80 and CD86 co-stimulatory molecules: most peripheral memory B-cells (SIgD−CD27^+^) co-expressed CD80 and CD86 in non-infected animals (**Table 2**). After infection, the percentages of both peripheral CD80^+^ and CD86^+^ memory B-cells decreased, reaching a nadir at D7 p.i. (1.8- and 1.9-fold decreases, respectively). A significant correlation was observed between the percentages of SIgD−CD27^+^ memory B-cells and those of SIgD−CD86^+^ (*Rho* = 0.93, *p*<0.003) or SIgD−CD80^+^ (*Rho* = 0.92, *p*<0.003) B-cells. In LN, most memory B-cells were either CD80^+^ or CD86^+^ in non-infected animals (*data not shown*). By D14 p.i., the percentage of CD80+ cells had decreased by a factor of 1.5, CD86+ had decreased by a factor of 1.2 and total memory B-cells by a factor of 1.3. At D21 and D28 p.i., the percentage of CD80^+^ had increased, approaching the baseline value, whereas the percentages of both CD86^+^ and total memory B-cells continued to decrease by similar amounts. The percentage of total SIgD−CD27^+^ memory B-cells was correlated with that of SIgD−CD86^+^ (*Rho* = 0.65, *p*<0.004) but not SIgD−CD80^+^ B-cells. In spleen, the percentage of CD80^+^ was lower than that of CD86^+^ B-cells within the memory B-cell subset, about 10% of memory B-cells were CD80+CD86+ (*data not shown*). After infection, the percentages of CD80 and CD86 memory B-cells had decreased by factors of 2.2 and 2 by D14 p.i., by factors of 1.6 and 2.3 by D21 p.i. and by factors of 3 and 3.5, respectively, by D28 p.i.. At the same time, the proportion of total memory B-cells decreased by factors of 2.3, 5.5 and 3.9 by D14, D21 and D28 p.i.. The percentage of total (SIgD−CD27^+^) memory B-cells was correlated with that of SIgD−CD86^+^ (*Rho* = 0.69, *p*<0.03) but not SIgD−CD80^+^ B-cells.

Consistent with the expression of CD95 by most memory B-cells in all organs, the percentage of total and CD95-expressing memory B-cells decreased concomitantly in SIV-infected animals (**Table 2**). A significant correlation was found between these percentages in blood (*Rho* = 0.98, *p*<0.002) and LN (*Rho* = 0.79, *p*<0.001) during acute SIV-infection.

Our data thus show a transient depletion of blood CD80^+^CD86^+^CD95^+^ memory B-cells without modification of the ratio between SIgM- and SIgG-expressing cells. In contrast, a preferential and sustained depletion of SIgG-, CD86-, CD95-expressing memory B-cells was observed in LN and spleen.

### SIV infection transiently affects B-cell survival, proliferation and Ig production

**Figure 2:**
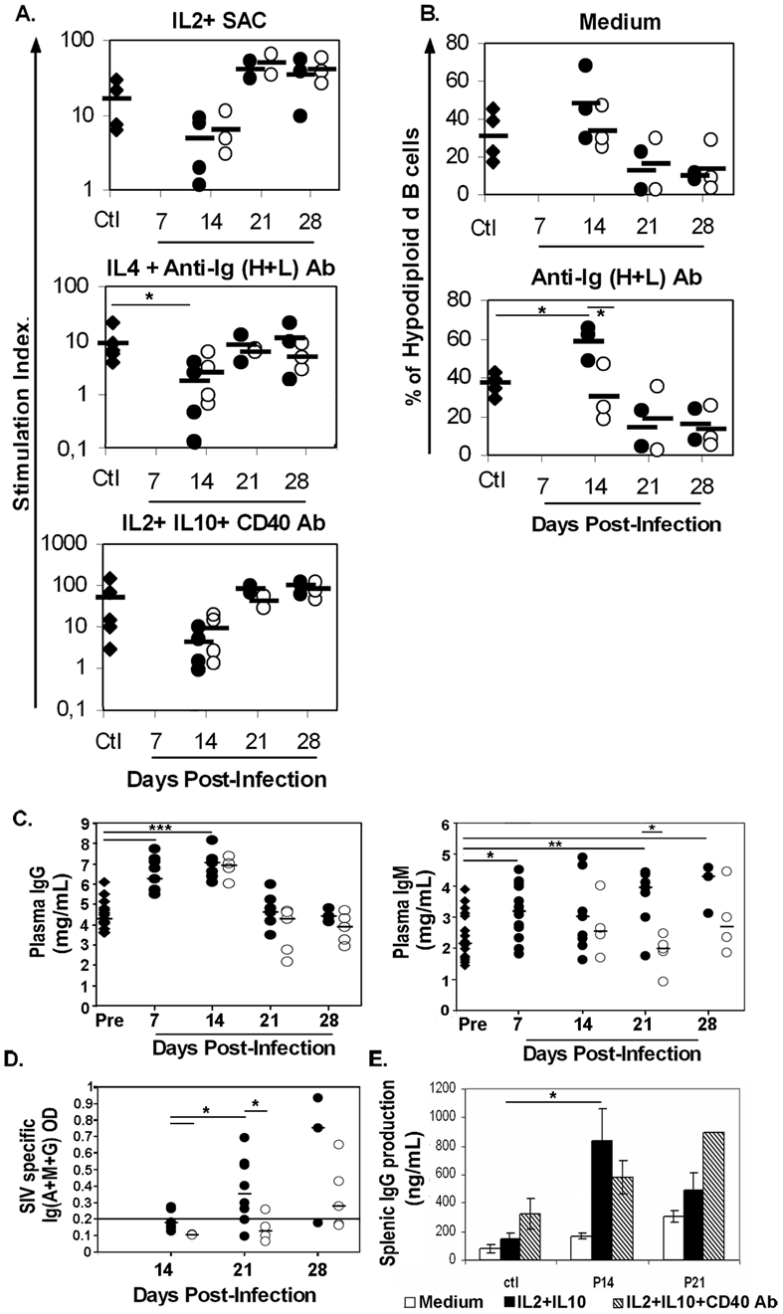
Functional impairment of B-cell response during early SIV infection. (A) Splenocytes were cultured with medium, IL2+SAC, IL4+anti-Ig (H+L) Ab or IL2+IL10+anti-CD40 mAb for three days. Proliferative response was evaluated in at least four non-infected animals (Ctl, *black diamond*), two (P21, H21), three (P28, H28) and four (P14, H14) SIV-infected animals. Each point represents the stimulation index (SI) for one animal. *Filled* and *open circles* represent P and H animals, respectively. Bars represent the mean SI in each group. SEM is less than 27%. *, *p* values<0.05 are shown. (B) Apoptotic cell percentages were determined in splenocyte cultures from four non-infected animals (Ctl, *black diamond*) and two (P21, H21, P28) or three (P14, H14, H28) SIV-infected animals. Each point represents the percentage of hypodiploid B-cells for one animal. *Filled* and *open circles* represent P and H animals, respectively. Bars represent the mean% in each group. SEM is less than 12%. *, *p* values<0.05 are shown. (C) IgG and IgM concentration were quantified in plasma taken before infection (Ctl, n = 14 for IgG and n = 16 for IgM,*diamond*). IgG concentrations were quantified in plasma from 10 (D7p.i.), 8 (D14p.i.), 7 (D21p.i.) and 3 (D28p.i.) placebo-treated animals (*Filled circle*) and from 4 (D14p.i.), 5 (D21p.i.) and 5 (D28p.i.) HAART-treated animals (*open circle*). IgM concentrations were quantified in plasma from 16 (D7p.i.), 9 (D14p.i.), 7 (D21p.i.) and 3 (D28p.i.) placebo-treated animals (*Filled circle*) and from 4 (D14p.i.), 5 (D21p.i.) and 4 (D28p.i.) HAART-treated animals (*open circle*). Median concentration is indicated for each group. Significant difference (*: *p*<0.05; **: p<0.01; ***: p<0.001) between values is indicated. (D) SIV-specific Ig (G+A+M) concentrations were measured in plasma from seven (D14p.i.), five (D21p.i.) and three (D28p.i.) placebo-treated animals (*Filled circle*) and from two (D14p.i.), four (D21p.i.) and five (D28p.i.) HAART-treated animals (*open circle*). Median OD value is indicated for each group *, significant difference (*p*<0.05) between values is indicated. (E) IgG production was measured in supernatants from spleen B-cell cultures of two non-infected animals and four (P14), or two (P21) SIV-infected animals. Results are mean values±SEM. *, *p* values<0.05 are shown

To assess whether the decrease in memory B-cells results from apoptosis, anergy or terminal differentiation, we investigated the effect of SIV infection on spleen B-cell function. The proliferation rate of SIV-infected spleen B-cells to B-cell receptor (BCR) stimulation was three (IL2+ SAC) and four (IL4+ anti-Ig H+L Ab) times lower than in non-infected animals at D14 p.i., but three and 1.4 times higher thereafter. Similarly, the response to CD40 stimulation was four times lower at D14p.i. and six and 8.4 times greater at D21 and D28 p.i., respectively (**Figure 2.A**). Levels of spontaneous and BCR-induced apoptosis were 20% higher in SIV-infected animals than in non-infected ones at D14p.i.. (**Figure 2.B**). Spontaneous and BCR-induced apoptosis at D21 and D28 p.i. were two and three times lower, respectively, than those observed in non-infected animals.

In addition to these effects on B-cell proliferation and apoptosis, a strong increase in plasma Ig concentration was observed in SIV-infected animals from D7 p.i.. However, the kinetics of IgG and IgM production differed (**Figure 2.C**): as compared to baseline values, IgM production was significantly increased by 29% up to D14 p.i. and by 54% to 69% thereafter, whereas IgG production was transiently increased by 50 to 60% by D14p.i. (p<0.001). Changes in IgG production were a direct result of an impaired B-cell response. Indeed, spontaneous and cytokine (IL2+IL10)-mediated IgG production by purified spleen B-cells was increased in SIV-infected animals at D14p.i. (**Figure 2.E**), probably reflecting the *in vivo* commitment to plasma cell differentiation. At D21p.i., IgG levels were still higher than control values in supernatants from spleen cells cultured with medium or stimulated with cytokines. The addition of CD40 mAb further enhanced cytokine-mediated IgG production in culture supernatants from non-infected and SIV-infected macaques on D21p.i. but not on D14p.i.. This suggests that available SIV-responsive B-cells are committed to plasma differentiation by D14p.i., whereas new SIV-responsive B-cells can be recruited into plasma cell differentiation from D21p.i.. Consistent with this notion, SIV-specific Ab were detected in only two out of seven plasma samples from D14p.i. but were present in most plasma samples from D21p.i. The median Ab plasma concentration thus significantly increased between D21 and D28 p.i. (**Figure 2.D**).

### Early HAART strongly decreases plasma viral load but not depletion of blood B-cells

**Figure 3:**
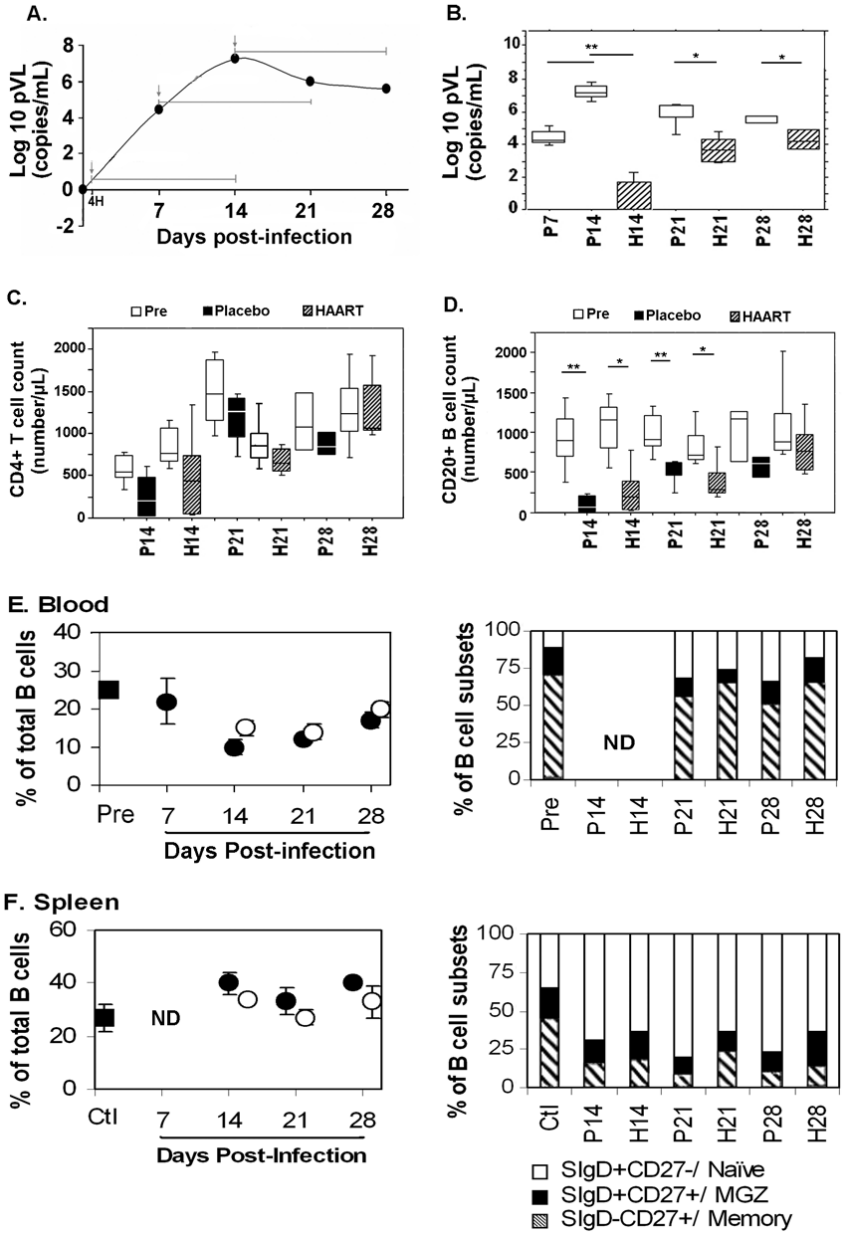
HAART has minimal effects on SIV-induced changes in B-cells. (A) The curve represents changes in plasma viral load (pVL, Log10copies/ml) in placebo-treated animals (median values) Arrows indicate the time of initiation of the two-week HAART treatment (4H, 7 or 14 days p.i.). Placebo and HAART-treated animals were therefore sacrificed at D14 p.i. (P14, H14), D21p.i. (P21, H21) and D28p.i. (P28, H28). (B) Plasma viral load (pVL, Log10copies/ml) at sacrifice in placebo- (*open symbol*) and HAART-treated (*hatched symbol*) animals are shown. Median value±IQ is indicated for each group. Significant differences (*: *p*<0.05; **: p<0.01) between groups are indicated. (C, D) CD4 T-cell (C) or B-cell (D) counts in placebo- (*black bars*) and HAART-treated (*hatched bars*) animals as compared to baseline values of the same animals before infection (*open bars*) are shown. Median value±IQ is indicated for each group. Significant differences (*: *p*<0.05; **: p<0.01) between groups are indicated. (E, F): *Left panels.* Percentages of blood B-cells before infection (E, pre, *black square*, n = 13), in non-infected animals (*black square* in F, n = 2 and G, n = 3), in placebo- (*black circle*, n = *3*) and HAART-treated (*open circles*, n = 3) SIV-infected animals. Mean percentage±SEM is indicated for each group. Non significant *p* values between placebo and HAART-treated groups are indicated. *Right panels.* The relative percentages of naive (SIgD+ CD27−, *open area*), memory (SIgD− CD27+, *hatched area*) and MGZ (SIgD+ CD27+, *black area*) B-cells were determined in controls (Ctl, 13 blood samples before exposure to SIV) for blood (E) or three non-infected animals for spleen (F).Three placebo- (P14, P21 and P28) or HAART-treated (H14, H21 and H28) SIV-infected animals were similarly investigated. Mean percentage±SEM is indicated for each group. ND: Not Done

To improve our understanding of the kinectics of B-cell dysfunctions during the acute phase of SIV infection, we compared data obtained in placebo- and HAART-treated macaques. Animals were subjected to HAART for two-weeks initiated at 4 h (beginning of the infection), 7 or 14 (peak of pVL) days p.i. and sacrificed at the end of HAART (i.e.: on D14 (H14), 21 (H21) and 28 (H28) p.i.) (**Figure 3.A**). At each time point, comparisons were done between placebo and HAART-treated groups infected for the same time period. A 10^6^ fold decrease in pVL was detected at D14 p.i. in H14 animals as compared to P14 animals whereas treatment initiated at D7 and D14 p.i. only led to 10^3^ fold (H21) and 10-fold (H28) decreases in pVL (**Figure 3.B**). Despite its efficiency in decreasing pVL in H14 animals, CD4 T-cell (**Figure 3.C**) and CD20 cell counts (**Figure 3.D**) were less than 10% higher in these animals than relevant placebo-treated animals. CD4+ T-cell and B-cell counts were correlated in HAART-treated animals (*Rho* = 0.925, *p*<0.0005).

The percentage of total blood B-cells was 20% and 10% higher in H14 and H21/28 animals respectively, than in placebo-treated animals (P14, 21 or 28). These differences were not statistically significant (**Figure 3.E** **, left panel**). However, percentages of memory and MGZ blood B-cells were partially restored at D21 and D28p.i.. In H28 animals, percentages of naive, MGZ and memory B-cells almost reached baseline values (**Figure 3.E** **right panel**). Only the difference between percentages of MGZ B-cells between P28 and H28 was statistically significant (*p<0.05*). The SIV-mediated increase in total spleen B-cells (**Figure 3.F** **, left panel**) was less marked in animals receiving HAART than in placebo-treated animals but loss of memory and MGZ B-cells was still prominent at D21 and D28 p.i. (**Figure 3.F** **, right panel**). The differences between percentages of total B-cells in placebo- and HAART-treated groups were not statistically significant but those between percentages of naive and memory B-cells on D21/28p.i. and of MGZ B-cells on D14p.i. were statistically significant (*p<0.05*).

Although HAART initiated at 4 h p.i. abrogated both spontaneous and BCR-induced apoptosis (**Figure 2.B**), the impairment in BCR- and CD40-mediated proliferation was only partially counteracted (**Figure 2.A**). Early HAART had no effect on the plasma IgG/M increase detected before D14p.i. (**Figure 2.C**), but led to a 50% decrease in IL2+IL10-induced IgG production *in vitro* (*data not shown*). HAART potently reduced SIV-specific Ab and total IgM production when initiated on D7 p.i. but was less efficient when initiated on D14p.i. (**Figure 2.C**).

### Changes in B-cell areas occur with different kinetics in mesenteric LN and spleen

**Figure 4:**
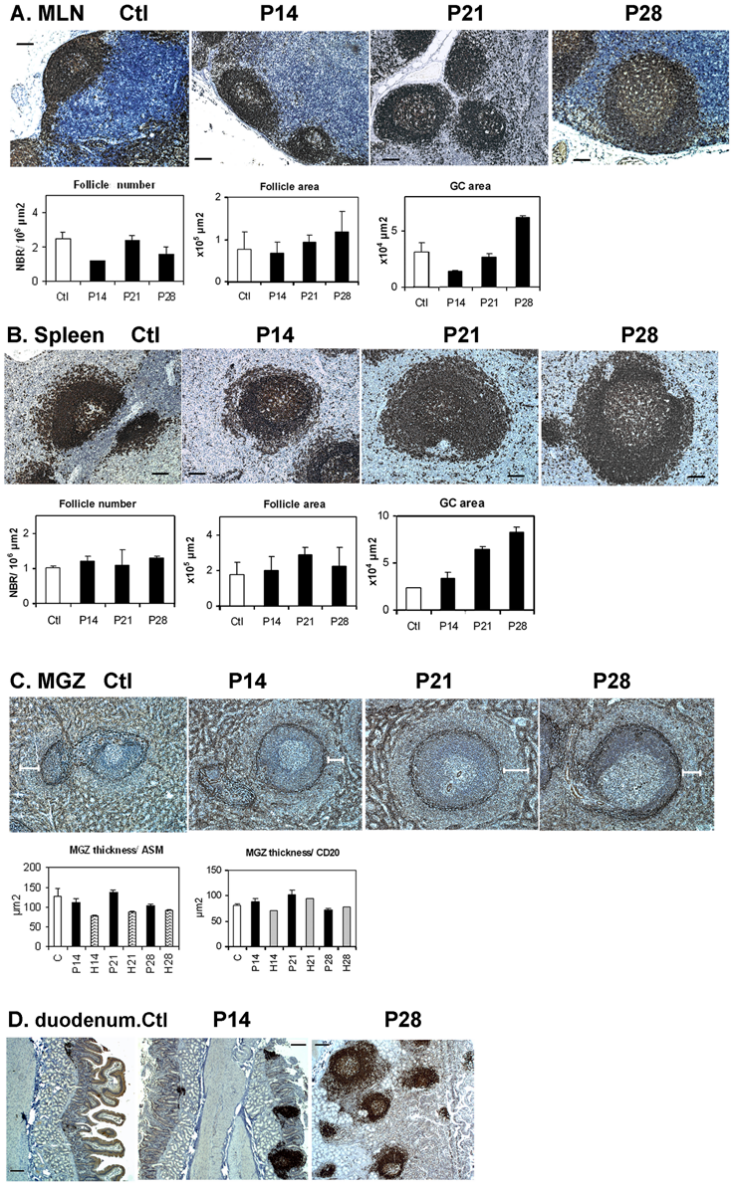
Changes in MGZ and B-cell areas in spleen and LN from SIV-infected animals. MLN (A) and spleen (B) sections from two non-infected (Ctl) or SIV-infected animals of each group (P14, P21, P28) were stained with CD20 mAb. Scale bars corresponding to 100 µm are shown in black. Staining from one representative animal of Ctl and P groups is shown. For each animal, the number of follicles was quantified for the whole section; results are expressed in number of follicles per 10^6^ µm^2^. The size of follicle and GC were quantified for 25±3 (MLN) and 34±3 (spleen) follicles per section. Mean values±SEM from two animals per group are shown. (C) Spleen sections from two non-infected (Ctl) or SIV-infected animals of each group (P14, P21, P28) were stained with ASM mAb for visualization of the MGZ. Scale bars corresponding to 100 µm are shown. Staining from one representative animal of Ctl and P groups is shown. For each animal, MGZ width was measured in 22±3 ASM-stained regions or 19±1 CD20-stained regions for each section. Mean values±SEM from two animals per group are shown. (D) Duodenum sections from one non-infected (Ctl) or two SIV-infected animals of P14 or P28 group were stained with CD20 mAb. Scale bars corresponding to 400 µm are shown. Staining from one representative animal of Ctl and P groups is shown.

To extend our data obtained from cell suspensions, we investigated the tissue organization in lymphoid organs of placebo- or HAART-treated SIV-infected animals. Spleen and mesenteric LN (MLN) taken on D14, 21 and 28 p.i. were analyzed by immunohistochemistry (IHC) with a large panel of primary antibodies (**supplemental** **Table S2**). The number of follicles and the size of GC were decreased in MLN on D14p.i. whereas the sizes of both follicles and GC were increased from D21 p.i.. (**Figure 4.A**). Although the numbers of spleen follicles were similar in non-infected and SIV-infected animals during the 28 days of infection, their size rapidly increased, peaking at D21p.i. (1.6- and 1.3-fold increase observed at D21 and D28p.i., respectively). The spleen GC size was 1.5 times greater at D14p.i.. and 3.6 times greater at D28p.i. than at baseline. (**Figure 4.B**). Changes in size of the spleen MGZ were analyzed after staining with CD20 (**Figure 4.B**), ASM (Alpha Smooth Muscle Actin) (**Figure 4.C**) or ICAM1 (*data not shown*) mAb. Measurement of the width of MGZ did not reveal any significant increase in size.

Whereas B-cell follicles were generally rare in the duodenum of non-infected animals, increasing numbers of B-cell follicles accumulated in this area in SIV-infected animals from D14p.i. (**Figure 4.D**). The size of these follicles also progressively increased and was frequently associated with well-developed GC from D28p.i.. Thus, trafficking to intestinal mucosa could transiently decrease the B-cell numbers in MLN before D14p.i. but this loss could be balanced by homeostatic mechanisms thereafter.

### Loss in follicular helper T-cells and infiltration of granzyme B^+^ and CD8^+^ cells in GC during acute SIV infection

**Figure 5:**
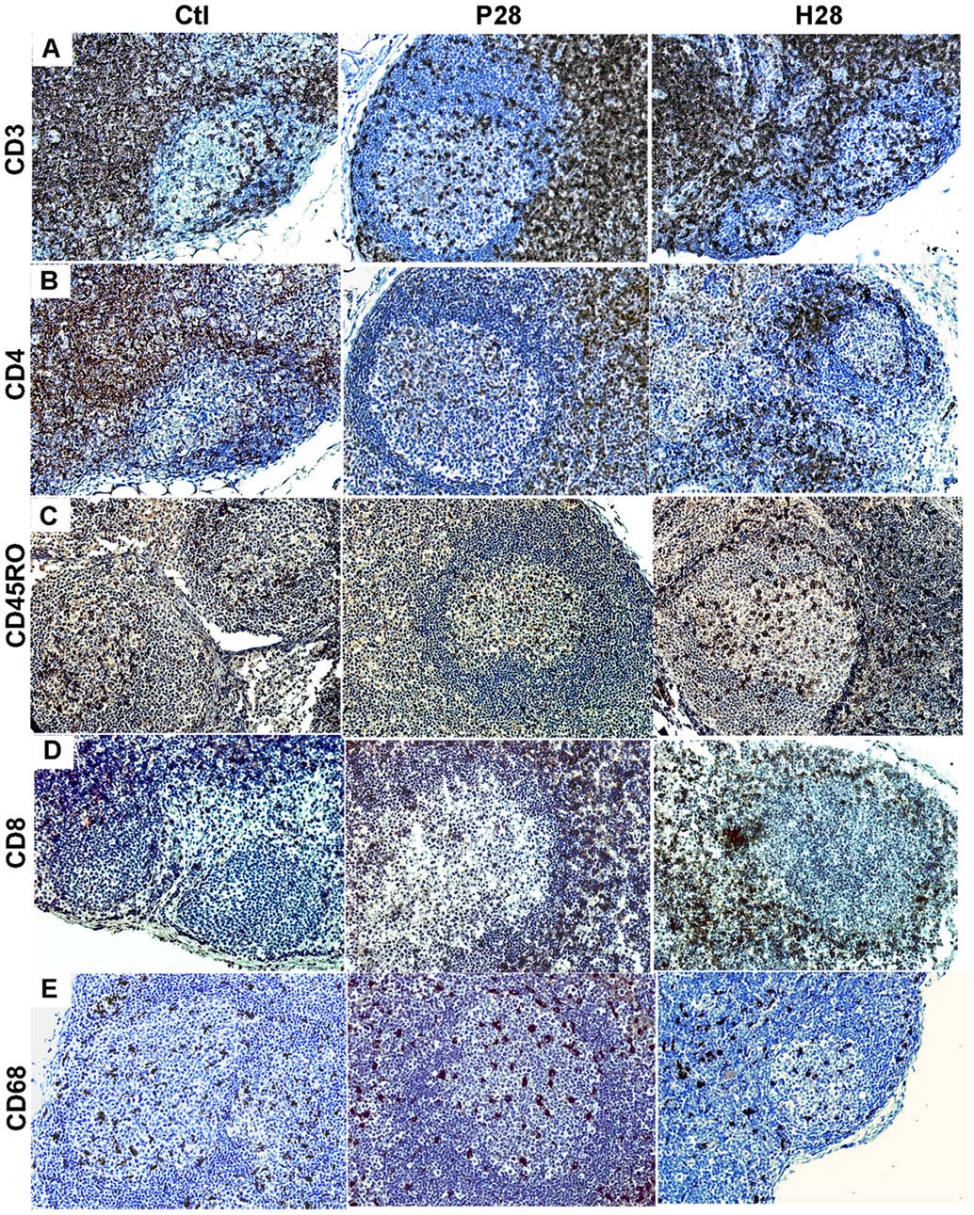
T-cell zone changes in placebo- and HAART-treated SIV-infected animals. MLN sections from two non-infected (Ctl), P28 and H28 animals were stained for CD3 (A), CD4 (B), CD45R0 (C), CD8 (D) and CD68 (E). Staining from one representative animal of each group is shown. Brown indicates positive staining; cell nuclei were counterstained blue by hematoxylin. Original magnification ×200 for all panels.

According to the loss in CD4^+^ T-cells during primary SIV/HIV-1 infection [29], [30] and the infiltration of cytotoxic CD8+ T-cells within the GC of various lymphoid organs in chronically infected individuals [12]–[14], we stained sections from spleen (*data not shown*) and MLN on D28 p.i. (**Figure 5**) for CD3, CD4, CD8 and CD45RO. We observed less intense staining with CD3 and CD4 mAb in the extrafollicular zone (EFZ) of placebo-treated animals than of non-infected animals, suggesting that there were fewer, positive cells. CD4 and CD3 staining on sections from HAART-treated animals appeared similar to that observed in non-infected animals. Due to their high number and low intensity staining, the number of CD4 T-cells present in the GC of non-infected and SIV-infected animals could not be quantified. However, we found that the number of CD45R0-positive cells in GC was 1.8 times lower in SIV-infected than in non-infected animals on day 28 p.i. (14±4.3 versus 25.4±8.3 cells per follicle, *p<0.05*, in SIV-infected and non-infected animals, respectively) (**Figure 5.C**), and two times lower in spleen (*data not shown*) suggesting a depletion in follicular helper T cells.

Although an increase in the number of CD8^+^ cells in the EFZ could be detected from D14p.i., infiltrating CD8^+^ cells were only detectable in GC from D21p.i. (**Figure 5.D**). The numbers of CD8^+^ cells were four (D21p.i.) and twenty (D28p.i.) times higher in the spleen of infected animals and five (D21p.i.) and 11 (D28p.i.) times higher in MLN (**Table 3**). We then examined their potential cytolytic activity by granzyme B (GrB) staining. This revealed increased numbers of positive cells in MLN and spleen from D14p.i., not only in EFZ but also in GC. In spleen and MLN, GC contained two to three times more GrB+ cells in placebo-treated animals than in control animals at D14 p.i. and about four times more at D28 p.i. (**Table 3**). However, the number of CD8+ cells was four times higher than that of GrB+ cells at D28p.i.. The mean number of CD8+ and GrB+ cells strongly decreased following HAART. The number of CD68 cells present in the EFZ zone and GC increased by a factor of two in spleen (24±11.5 *vs* 13.1±7. cells on D28 p.i., *p<0.0001*) but remained similar in MLN (21.3±9.7 *vs* 19.2±7.3, on D28p.i., non-significant *p* value) (**Figure 5.E**).

**Table 3:** CD8 and Granzyme B cells infiltrate GC of SIV-infected animals.

Cell number/GC				
	Spleen		LN	
	CD8+ cells	Granzyme B cells	CD8+ cells	Granzyme B cells
Ctl^a^	<1	<1	<1	<1
P14	<1	2.7±1.3^**^	<1	1.6±1.4^*^
H14	<1	0.5±0.7^••^	<1	0.1±0.3^•^
P21	4.0±0.3^**^	2.8±2.5^**^	5.1±1.6^**^	3.6±2.3^**^
H21	1.3±0.5^•^	1.6±1.2	1.3±0.9^•^	0.6±1.0
P28	20.2±1.6^**^	4.1±1.4^***^	11.2±1.2^***^	4.3±2.2^*^
H28	5.2±3.0^•^	0.8±0.7^••^	3.2±1.7^••^	0.6±0.7

a Ctl as control for non-infected animals.

Results are mean±SEM; n = 2 animals.

8±2 GC per animal were analyzed.

^*^p<0.05; ^**^ p<0.01; ^***^ p<0.001: Ctl vs. placebo-treated macaques.

^•^p<0.05; ^••^ p<0.01; ^•••^ p<0.001: placebo- vs. HAART-treated macaques.

### Changes in GC organization and plasma cells during acute SIV infection

**Figure 6:**
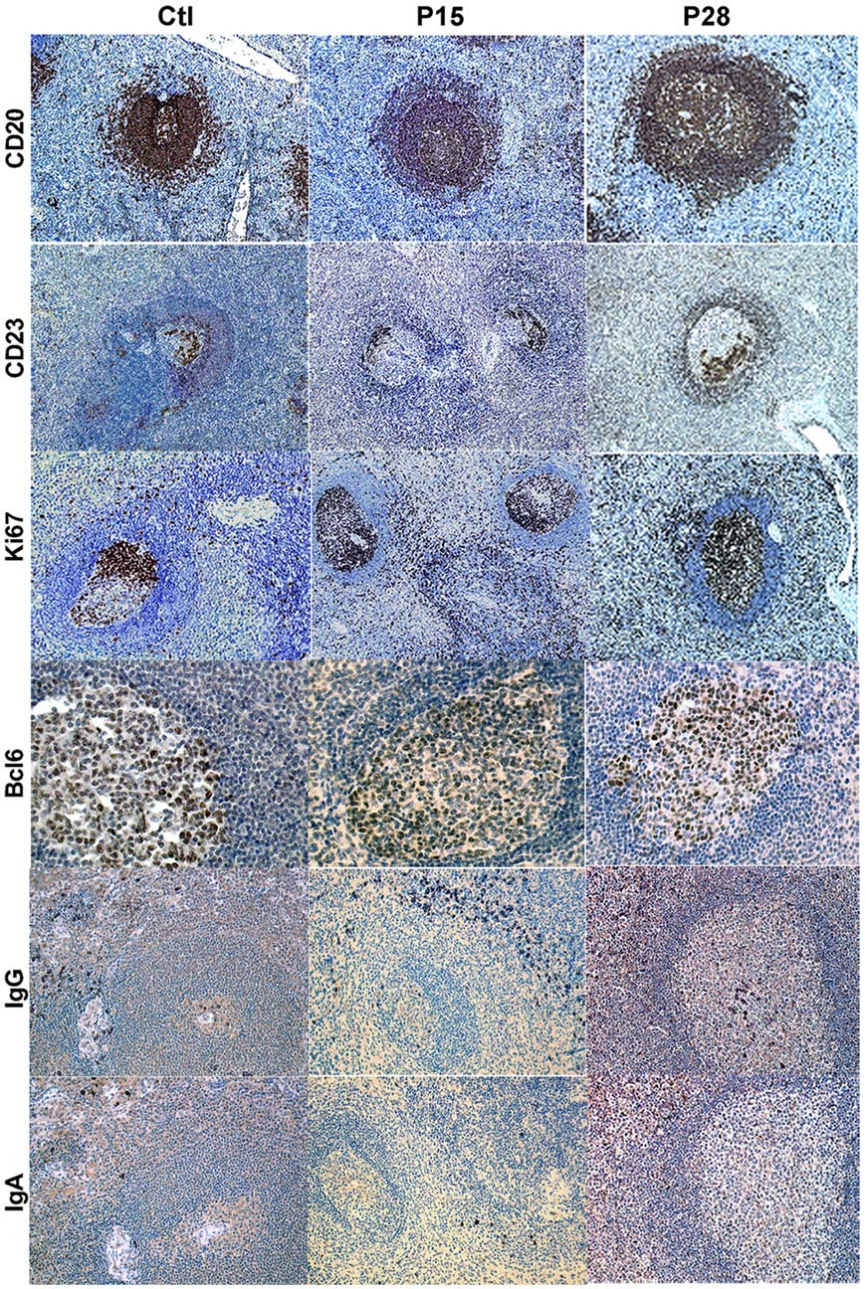
Progressive changes within splenic B-cell areas from placebo treated SIV-infected animals on D14 and D28p.i. Spleen sections from two non-infected (Ctl) and two P28 SIV-infected animals were stained for CD20 (A), CD23 (B), Ki67 (C), Bcl6 (D), IgG (E) and IgA (F) expression.Staining from one representative animal of each group is shown. Brown indicates positive staining; cell nuclei were counterstained in blue by haematoxylin. Original magnification ×100 (A–C), ×400 (D) and 200× (E–F).

**Figure 7:**
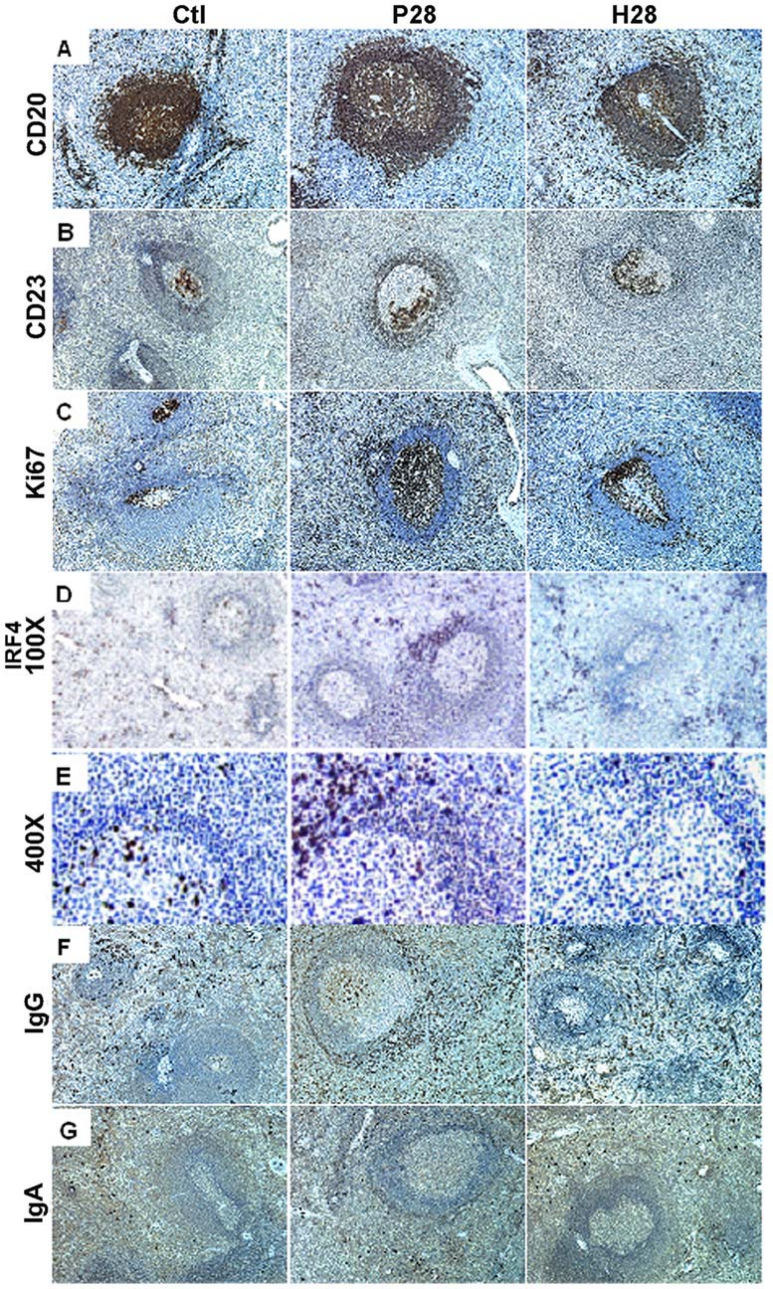
Phenotypic change within splenic B-cell areas from placebo- and HAART-treated SIV-infected animals. Spleen sections from two non-infected (Ctl) and two P28 and H28 SIV-infected animals were stained for CD20 (A), CD23 (B), Ki67 (C), IRF4 (100× and 400×) (D, E), IgG (F) and IgA (G) expression. Staining from one representative animal of each group is shown. Brown indicates positive staining; cell nuclei were counterstained in blue by haematoxylin. Original magnification ×100 for all but panel E.

As described above, the sizes of total (CD20 staining) and GC (staining with Bcl6) B cell-populated areas were already increased on D14p.i. and even more at D28p.i. in spleen (**Figure 6**) and MLN (**supplemental** **Figure S3**). In contrast, the mantle zone was thinner in SIV-infected animals than in controls on D28p.i.. Several other striking differences between control and SIV-infected animals were observed from D14p.i. on: (i) a strong increase in Ki67+ proliferating cells, with Ki67 staining delineating the dark zone (DZ) of GC in non-infected individuals; (ii) a strong increase of Ki67+ cells in EFZ; (iii) reorganization of the follicular dendritic cell (FDC) network, revealed by staining with CD23 or ICAM1 (*data not shown*). CD23 staining was distributed throughout the GC of control animals, but was frequently patchy, with preferential staining at one side of GC, probably in the light zone (LZ) in placebo-treated animals. These observations are indicative of an impaired expansion of the FDC network and LZ during early SIV infection. HAART significantly reduced these alterations (**Figure 7** **and supplemental** **Figure S2**).

In the absence of appropriate CD138 and CD38 mAb for IHC analysis in cynomolgus macaques, we used IRF4, IgG and -IgA Ab to detect plasma cells. (**Figure 6**). An increase in the number of IRF4-positive cells was detected in GC and EFZ (including red pulp) in spleen on D14. and D28p.i. (**Figure 6**). From D14p.i.on, increased numbers of IgG, but not IgA, plasma cells were observed in EFZ, including spleen red pulp. The number of IgG plasma cells within the GC was similar in control and non-infected animals on D14p.i. but was increased from D21p.i. on in spleen and at D28p.i. in MLN. The number of IgG plasma cells in GC at D28p.i. was increased by a factor of four in spleen and six in MLN of SIV-infected animals. No IgA plasma cells were detected in the GC of spleen (**Figure 6**) or MLN (*data not shown*) whereas they were present in similar proportions in the extrafollicular zones of the spleen (**Figure 6**) or MLN (*data not shown*). HAART significantly reduced these alterations (**Figure 7** **and supplemental** **Figure S2**).

## Discussion

Impaired production of neutralizing HIV-specific antibodies during natural exposure to virus or during vaccination protocols is one major hallmark of HIV infection [15], [31]. Even long-term HAART is only partially efficient in preserving the humoral response in HIV^+^ patients, suggesting that alternative immune-modulators are required. Given that the nature of the events taking place during primary infection is critical for initiation of antiviral responses and predictive of long-term disease progression, a better understanding of the early pathogenic effects of HIV on B-cells is needed. Since early time points of acute HIV infections are very difficult to study in humans, we specifically examined changes in B-cell subsets from several lymphoid organs during the acute phase of the disease in SIV_mac251_-infected cynomolgus macaques. Our data reveal a transient peripheral B-cell lymphopenia (count and percentage) with a nadir at D14p.i., coinciding with the plasma viremia peak and the nadir in NK and CD4^+^ or CD8^+^ T-cells. Similar panleukopenia is observed during the acute phase of most viral infections in primates and mice.

In contrast to blood, the percentage of total B-cells increased in spleen from D14p.i. despite few changes in the percentage of T-cells in spleen before D21p.i. (*data not shown*). During the same time, the percentage of total B-cells did not vary in peripheral LN (axillary and inguinal LN) whereas a consistent influx of B-cells into the small intestine occurs from D14p.i. with the number and size of follicles progressively increased between D14 and D28p.i.. Altogether these data suggest that blood B-cells traffick to lymphoid organs but preferentially accumulate in the spleen and the small intestine. A drop in blood B-cells associated with a selective tissue homing has previously been reported in mice acutely infected by various pathogens [32]–[34]. Local sequestration of B-cells in these mice correlated with type I IFN-induced up-regulation of CD69. In these murine infection models, membrane CD69 interferes with surface expression of sphingosine 1-phosphate receptor 1 and blocks B-cell egress from particular lymphoid organs [34], [35]. Consistent with the B-cell hyper-responsiveness to CXCL13 observed in HIV-infected patients [9], a similar mechanism may underlie B-cell accumulation in spleen follicles and MGZ during acute SIV infection. Indeed, early peaks of type I IFN and TNFα are detectable in plasma during HIV/SIV infection, before or at the same time as the viremia peak [24], [36]–[39]

Regardless of changes in total B-cells, a selective loss in CD27^+^ memory B-cells occurred in blood, LN and spleen. This loss was also demonstrated by decreases in CD80-, 86- and 95-expressing B-cells in these three organs. The decrease in circulating memory B-cells was transient, correlating with that of total B-cells, and occurred without any change in the ratio of SIgM^+^/SIgG^+^ memory B-cells. This suggests that this decrease mainly resulted from B-cell trafficking to lymphoid organs, where a B-cell response is initiated. The relative percentage and number of memory B-cells increased in blood from D21p.i., suggesting that memory B-cells are newly formed or are sent back into circulation from tissues. This coincides with reduced levels of inflammatory cytokines in plasma, observed in SIV-infected macaques after 2 weeks p.i. [36], [40]. Given previous findings in primary HIV-infected patients [15], the blood B-cell repertoire in SIV-infected macaques must undergo significant changes at four weeks p.i. and probably also later on. This hypothesis remains to be verified in SIV-infected macaques previously vaccinated with recall antigens.

Memory B-cells in spleen or LN steady declined until D28p.i., correlating with a preferential depletion in SIgG-expressing memory B-cells. This cell loss peaked at D14p.i. in spleen but was more progressive in LN. This loss may have been initiated by SIV-induced B-cell apoptosis by D14p.i. and early commitment to terminal differentiation. Most of the IgG molecules produced *in vitro* were non-specific for SIV, with SIV Ab remaining undetectable in culture supernatants (*data not shown*). Given that HAART initiated 4 h p.i. fully prevented apoptosis but partially reduced *in vitro* IgG production, independent control mechanisms may be involved. Previous data obtained in SIV- or HIV-infected individuals suggest that apoptosis is induced by various virus-induced signals [41], [42], whereas Ig production is rather promoted by increased cytokine production [43], [44].

Plasma IgG and IgM levels were increased by D14p.i., whereas SIV-specific antibodies were detectable only from D21p.i. on. Therefore, we suggest that an early T-independent response takes place before D14p.i.. In humans, unlike in mice, MGZ contains both CD27^+^SIgM^+^SIgD^+^ MGZ and CD27^+^ SIgD−SIgG^+^ memory B-cells poised to differentiate rapidly into plasma cells in response to T-independent signals, including BAFF, APRIL and IL21 [45]. Elevated BAFF plasma levels have only been reported in chronically HIV-infected patients with a low CD4 count [46], [47]. Inflammatory cytokines and CD40 ligand are well-established inducers of BAFF production by monocytes, macrophages and dendritic cells [48] and, moreover, He et al have recently shown that gp120-induced production of BAFF by monocytes enhances the polyclonal IgG and IgA production by a subset of gp120-activated MGZ-like B-cells [19]. BAFF production may therefore be triggered during acute SIV-infection and contributes to the early, potentially T-independent increase in plasma IgG/M. Although the histological size of MGZ did not significantly vary, the frequency of (CD27^+^SIgD^+^) MGZ B-cells decreased transiently in LN between D14 and D21p.i. and steadily in spleen and blood from D14p.i. which could be due to their commitment to T-independent terminal differentiation. Similarly, a loss in IgM-only memory B-cells, associated withan impaired response to T-independent antigens has been reported in primary and chronically HIV-infected patients [15], [17] whereas a splenic MGZ hypoplasia was reported only in chronically HIV-infected patients [12]. An increase in IgG-expressing plasma cells was observed in the EFZ of spleen and LN from D14p.i.. The fact that HAART had a moderate effect on increased plasma IgG/M by D14p.i. suggests that this increase was independent of viral replication *per se* but is correlated with SIV-induced inflammatory signals, including BAFF as also suggested by the work of He et al [19]

IgG-expressing plasma cells were only detectable in GC from D21p.i.. The SIV-specific response was therefore likely to have been initiated between D14 and D21p.i.. Initiation of the specific response is likely to be delayed until viral antigens are locally available in sufficient amounts. Consistent with a recent study by Cinamom, showing that MGZ B-cells transport immune complexes into the GC [11], [49], shuttling of MGZ B-cells to follicles could enhance the SIV-specific Ab response. According to recent data in HIV-infected patients, hyper-responsiveness to CXCL13 might favour the homing of MGZ B-cells into GC [9]. The marked and concomitant decrease in SIV-specific Ab and plasma IgM observed after D14p.i. in HAART-treated animals is consistent with SIV-specific Ab being essentially IgM until D28p.i.. Although IgM in nature, these early Ab would be protective and probably play an essential role in generating immune complexes to be transported into GC [49]. Viral particles, through interactions between trimeric gp120 and B-cells, could trigger a T-independent, SIV non-specific Ab response before D14p.i., predominantly targeting gp120-associated carbohydrates. As viral replication progresses, a T-dependent response might occur. Similar conclusions have been drawn by Trkola *et al*. in chronically infected patients subjected to short-term interruption of treatment [50]. Alternatively, soluble Nef present in GC of HIV-infected patients might inhibit CD40-dependent Ig-switching of GC B-cells [51]. The immunosuppressive effect of Nef on the B-cell response to SIV was previously suggested by the work of Chakrabarti et al, who showed that Nef-deleted SIV strain induced a more rapid development of GC and circulating SIV-specific Ab than pathogenic SIV strain [52]. Therefore, soluble Nef is likely to contribute to the delayed SIV-specfic Ab response observed during the acute phase of infection.

GC hyperplasia was detected in spleen and MLN of SIV-infected animals from D14 and D21p.i., respectively. The less intense staining observed in spleen and MLN suggests that fewer CD4+ T-cells may be present in EFZ of infected animals than in non-infected ones. However, the distribution of CD4+ cells in GC of infected animals was similar to those observed in non-infected animals before D28p.i.. This contrasts with the impaired polarization of GC observed in chronically infected patients [13]. A decrease in CD45R0 cells, likely CD4 follicular helper T-cells, was detected within GC on D28p.i.. Consistent with their activated/memory phenotype, SIV infection might lead to their apoptosis *in situ* or prevent their homing to GC. In contrast, infiltrated GrB+ cells and CD8+ cells were present in GC from D14p.i. and D21p.i. respectively. Given previous findings in chronically infected macaques [53] or patients [13], [54], it seems likely that these cells are cytolytic T-cells involved in virus control. Similarly, GC of SIV-infected animals contained more Ki67+ positive cells, demonstrating an expanded DZ, whereas follicular dendritic cells (FDC) had a patchy distribution localized to one edge of GC. The numbers of total plasma cells (Vs38c, IRF4) and IgG-expressing plasma cells increased in the red pulp, EFZ and GC of SIV-infected animals, whereas IgA-expressing cells were present in similar proportions in the red pulp of non-infected and SIV-infected animals. In spleen, most of these changes were fully or partially (plasma cells) cured by HAART, no matter when HAART was initiated. HAART did not prevent the decrease in GC size in MLN, observed at D14p.i., but cured the increase in GC and other changes observed at D28p.i.. This dual effect is mainly independent of virus replication and is probably sustained by inflammatory signals.

In conclusion, we have combined IHC studies of lymphoid organs with phenotypic and functional analyses of B-cells to further elucidate the mechanisms underlying B-cell dysfunction during acute SIV infection. Consistent with unresponsiveness to early HAART, we suggest that T-independent signals, possibly triggered by direct contact with virions and relayed by inflammatory cytokines, are important in early B-cell dysfunction—a major feature of the SIV/HIV-specific B-cell response strongly favoring non-specific Ig production. A better understanding of the inflammatory signals acting on B-cell subsets should help to design of new therapeutic strategies, potentially delaying the initiation of HAART which strongly decreases the HIV/SIV humoral response.

## Materials and Methods

### Animals, virus and treatment and plasma viral load

Thirty adult male cynomolgus macaques (*Macaca fascicularis*) were inoculated intravenously (i.v) with 50 AID50 of the pathogenic SIVmac251 strain, as previously described [23]. Animals were housed and cared for in accordance with European Guidelines for animal care. All protocols used in this study were reviewed and approved by an regional animal care and use committee. Three groups of five animals were killed on days (D) 14, 21 or 28 post-infection (p.i.) respectively; these were placebo-treated groups P14, P21 and P28. The three other groups of five animals initiated a two-week HAART from 4 h, 7 or 14 days p.i.. HAART was a combination of 4.5 mg/kg 3-azido-2,3-dideoxythymidine (AZT), 2.5 mg/kg 2,3-dideoxy-3-thiacytidine (3TC), and 20 mg/kg indinavir given twice daily, via the oral route, as previously described [23]. These animals were sacrified at the end of HAARTand referred to as HAART-treated groups H14, H21 and H28, accordingly. All procedures with animals were performed after general anesthesia with ketamine chlorhydrate (Rhone-Merieux, Lyon, France). Plasma viral load was determined by real-time PCR, as previously described [55]. Non-infected animals were used as controls.

### Mononuclear cell counts and phenotypes

PBMC were obtained from peripheral blood on BD Vacutainer® CPT™. Absolute numbers of leukocytes (cells/µl) were obtained by analysis of 15 µl of blood, collected on EDTA, in Micro Diff II (Beckman Coulter, Fullerton, USA). B-cells (CD20), monocytes (CD14), total T (CD3), CD4, CD8 cells and NK (CD3−CD8+) cells were quantified by flow cytometry with 50 µl of titrated combinations of fluorochrome–conjugated mAbs added to 100 µl of whole blood, as previously described [55]. After incubation for 15 min at 20°C in the dark, cells were subjected to red blood-cell lysis and were then post-fixed with 300 µl of Cell Fix 1× (BD Biosciences) and kept at 4°C in the dark. Surface marker expression was analyzed with a LSR I flow cytometer and by FlowJo software (Tree Star Inc., Ashland, OR). Similar combinations of mAb were used on isolated lymph node mononuclear cells and splenocytes to determine the percentage of each lymphoid subset.

### Immunophenotyping of B-cell subsets in blood, LN and spleen

Spleen, axillary and inguinal LN were collected from SIV-infected animals and four non-infected ones as controls. Splenocytes and LN mononuclear cells were obtained by mechanical disruption, passed through nylon mesh cell strainers with 40-µm pores and further purified by Ficoll gradient centrifugation (BD Biosciences, Franklin lakes, NJ). Freshly isolated cells were subjected to phenotypic analysis or kept at −80°C until use. Blood, spleen and LN B-cell subsets were identified by three-colour flow cytometry detecting the combined expression of CD20 with CD27, CD80, CD86, CD95, surface IgD (SIgD), SIgM or SIgG. Briefly, cells were stained with 50 µl of titrated combinations of fluorochrome–conjugated mAbs listed in **supplemental** **Table S1**. Data were analyzed as described above.

### Isolation of spleen B-cells and functional assays

Spleen B-cells were purified using PE-conjugated CD20 mAb (L27, BD Biosciences) and PE-conjugated magnetic beads (EasySep, StemCell, Vancouver, Canada). The cell suspension was incubated with CD32 mAb (2E1, Beckman Coulter) before CD20-PE addition according to the manufacturer's instructions. Only B-cell fractions that contained ≥80% CD20+ cells were used for functional studies. Splenic B-cells were cultured in RPMI 1640-glutamax medium supplemented with 1 mM sodium pyruvate, 100 µg/ml streptomycin, 100 U/ml penicillin, 10 mM HEPES buffer, 2 mM non-essential amino acids (all from Invitrogen, Carlsbad, CA) and 2% (proliferation) or 10% (apoptosis and IgG production) heat-inactivated FCS (PAA laboratories GmbH, Les Mureaux, France).

#### Apoptosis

Splenocytes were cultured in triplicate with 25 µg/ml F(ab')_2_ rabbit anti-human Ig(H+L) Ab (Jackson ImmunoResearch Laboratories, West Grove, PA) or medium (spontaneous apoptosis) for 24 h. After several washes, cells were fixed and permeabilized in 70% ethanol overnight, at 4°C. Cells were then treated for 30 min at 37°C with 100 µg/ml RNAse A (Sigma, St Louis, MO) and labelled with DAPI (Molecular Probes, Invitrogen) for 15 min at 20°C and analyzed by flow cytometry. Apoptotic cells were defined as hypodiploid cells.

#### Cell proliferation

Splenocytes were cultured in triplicate with 25 µg/ml F(ab')_2_ rabbit anti-human Ig(H+L) Ab (Jackson ImmunoResearch Laboratories) and 20 ng/ml IL4 (R&D systems, Abingdon, UK), *Staphylococcus Aureus* cowan I (1/10.000, Calbiochem, La Jolla, CA) and 20 ng/ml IL2 or 10 µg/ml CD40 mAb (G28.5) plus 20 ng/ml IL2 and 50 ng/ml IL10 (both cytokines from R&D systems) for 72 h. Proliferative responses were measured by pulsing cultures with 1 µCi per well [*methyl*-3H] thymidine (Amersham, les Ulis, France) for the last 12 h of the culture. Results are cpm means±SEM of triplicate measurements. Stimulation index (SI) was calculated as the ratio between the cpm means for cultures with and without stimuli.

#### Ig quantification

Splenocytes were cultured for 10 days at 37°C with medium, 20 ng/ml IL2 plus 50 ng/ml IL10, with or without 10 µg/ml CD40 mAb. Ig concentrations were determined in cell-free culture supernatants or in plasma by specific ELISA. Rabbit anti-human IgG+M+A, HRP-conjugated goat anti-rhesus IgM (μ chain-specific) (both from Nordic Immunological Laboratories, Tilburg, The Netherlands) and rhesus IgM (Gentaur, Brussels, The Netherlands) were used for IgM quantification. Goat anti-monkey IgG, HRP-conjugated goat anti-rhesus IgG (both from AbD Serotec, Oxford, UK) and rhesus IgG (Rockland, Gilberstville, PA) were used for IgG quantification. Results are expressed as mean concentration (mg/ml; ±SEM) of duplicate measurements.

#### Anti-SIV Ab

Specific anti-SIV Ab were detected in plasma by ELISA using Genscreen HIV1/2 kit, version2 (Bio-Rad Laboratories, Redmond, WA). Results are expressed in OD obtained from A_450_ nm readings.

### Immunohistochemistry (IHC) and quantitative image analysis

#### Paraffin-embedded tissues

IHC were performed on deparaffinized spleen, MLN or small intestine sections, with primary mAb (**supplemental** **Table S2**). Ab binding was visualized with the StreptABComplex/HRP duet kit and DAB (3,3 Diaminobenzidine) (Dako). Slides were counterstained with Mayer's hematoxylin and mounted in permanent mounting media (Dako).

#### Frozen tissues

MLN and spleen biopsies were frozen and 5-µm cryostat sections were fixed in acetone, blocked in 1% BSA and stained with CD8 mAb (**supplemental** **Table S2**). Ab binding was visualized with StreptABComplex/HRP duet kit and DAB. Slides were counterstained with Mayer's hematoxylin and mounted in permanent mounting media (Dako).

Images from spleen and MLN sections were obtained with a light microscope Zeiss (Laboandco, Mandres-les-Roses, France), captured by a Microfire microscope camera system (Optronics, Goleta, CA) and analyzed with Mercator 4.42 software (ExploraNova, La Rochelle, France).

### Statistical analysis

Data are expressed as mean±SEM, unless otherwise indicated. Non-parametric analysis (Wilcoxon signed-rank test and Mann-Whitney test) and correlations (Spearman's rank coefficient) were performed using StatView software (SAS Institute, Cary, NC). *p* value≤0.05 was considered as significant.

## Supporting Information

Table S1 Antibodies used in FCM
(0.28 MB DOC)

Table S2 Antibodies used in IHC
(0.23 MB DOC)

Figure S1 Correlation between plasma viral load and blood T- or B-cell counts during SIV infection. Correlation between plasma viral load (pVL, Log10copies/ml) and blood CD4 (A), CD8 (B) or CD20 (C) cell count (cells/µl) in SIV-infected animals is shown. Correlation between blood B-cell count and percentage (D) or CD4 cell count (E) in SIV-infected animals is shown. Statistical significance was assessed by Spearman's rank correlation test; Rho and p values are indicated.
(0.38 MB TIF)

Figure S2 Phenotypic change within MLN B-cell areas from placebo- and HAART-treated SIV-infected animals. MLN sections from two non-infected (Ctl), P28 and H28 animals were stained for CD20 (A), CD23 (B), Ki67 (C), Vs38c (D) and IgG (E) expression. Staining from one representative animal of each group is shown. Brown indicates positive staining; cell nuclei were counterstained in blue by hematoxylin. Original magnification ×100 for A to E and ×200 for C.
(8.77 MB TIF)

Figure S3 Phenotype change within mantle zone of follicles from placebo and HAART-treated SIV-infected animals. Spleen sections from two non-infected (Ctl), P28 and H28 animals were stained for CD3 (A) and CD68 (B) expression. Staining from one representative animal of each group is shown. Brown indicates positive staining; cell nuclei were counterstained in blue by hematoxylin. Original magnification 100× and 400× for both markers.
(4.49 MB TIF)
