# Supplementary figures and images for: Tissue-Specific B-Cell Dysfunction and Generalized Memory B-Cell Loss during Acute SIV Infection
Source: PLoS One. 2009 Jun 19;4(6):e5966. doi: 10.1371/journal.pone.0005966 (PMC2695011; doi:10.1371/journal.pone.0005966)

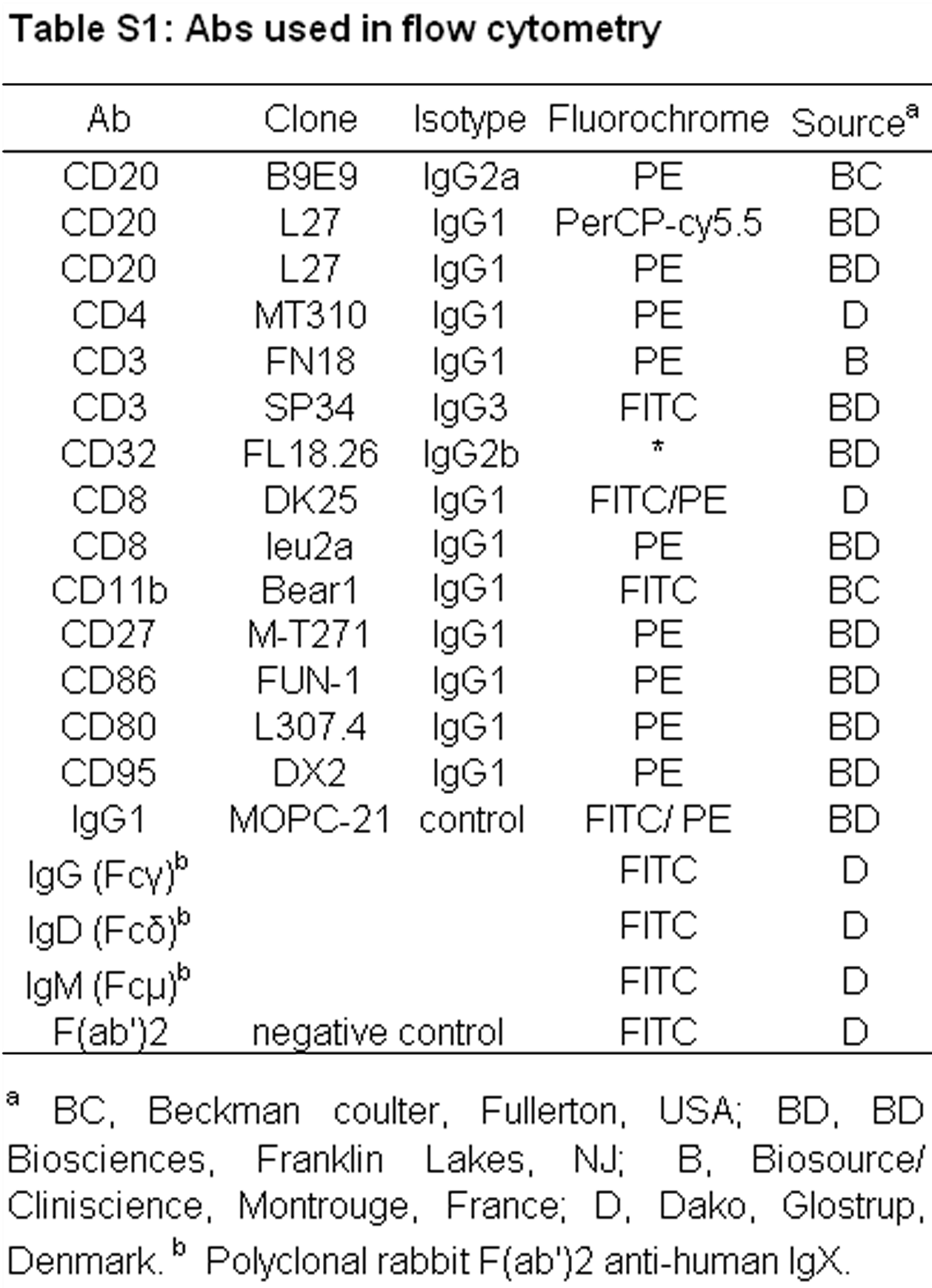

Supplement: Table S1 — Antibodies used in FCM (0.28 MB DOC) [file pone.0005966.s001.doc]

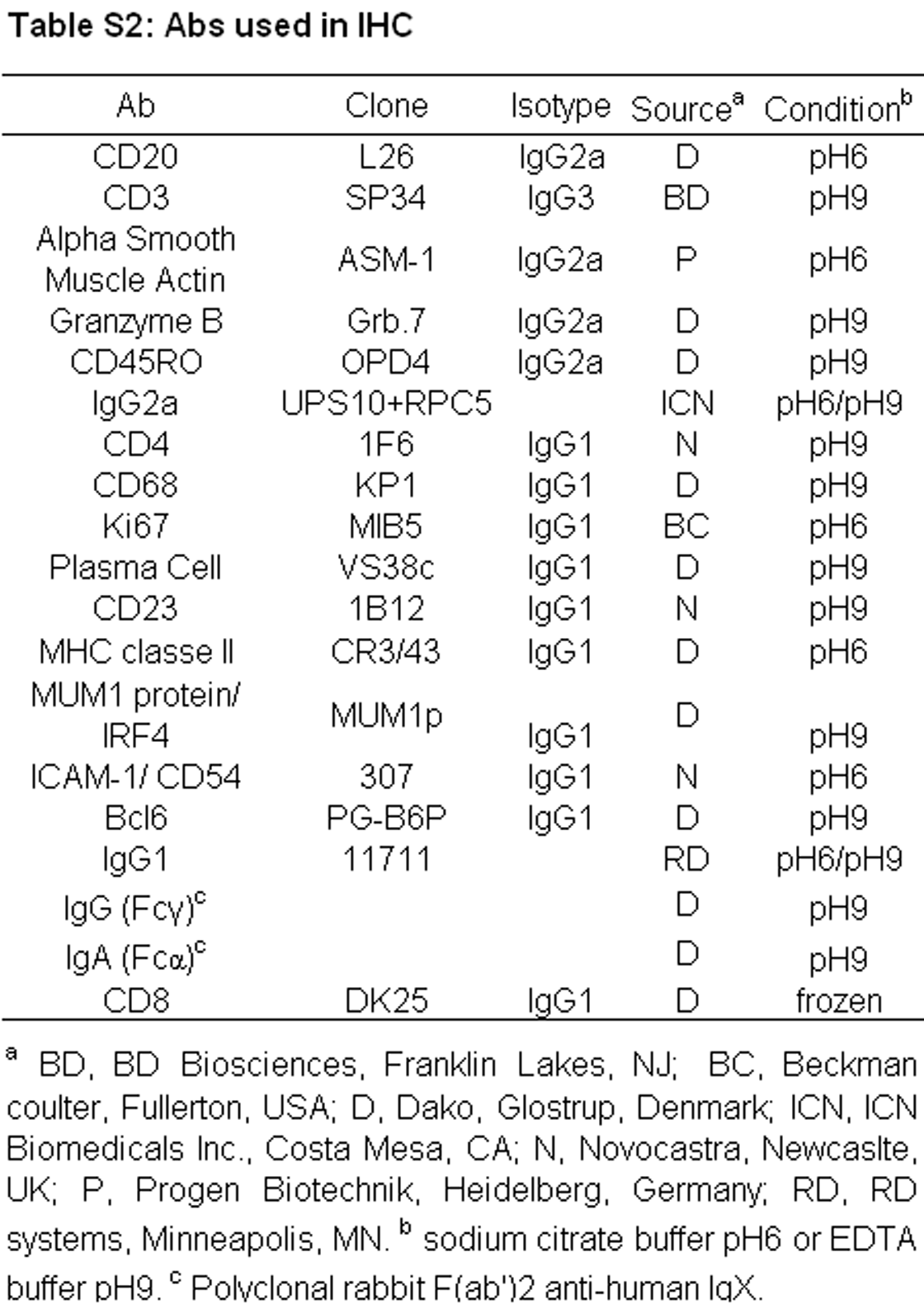

Supplement: Table S2 — Antibodies used in IHC (0.23 MB DOC) [file pone.0005966.s002.doc]

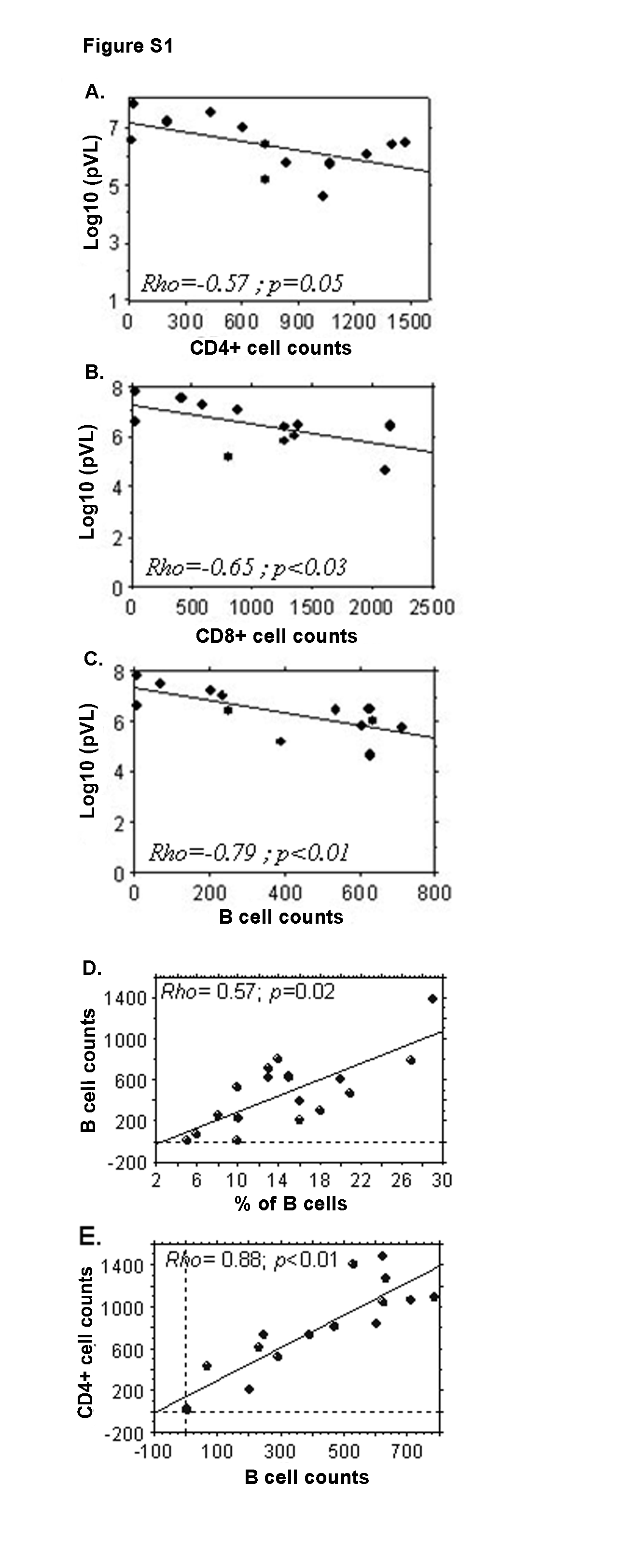

Supplement: Figure S1 — Correlation between plasma viral load and blood T- or B-cell counts during SIV infection. Correlation between plasma viral load (pVL, Log10copies/ml) and blood CD4 (A), CD8 (B) or CD20 (C) cell count (cells/µl) in SIV-infected animals is shown. Correlation between blood B-cell count and percentage (D) or CD4 cell count (E) in SIV-infected animals is shown. Statistical significance was assessed by Spearman's rank correlation test; Rho and p values are indicated. (0.38 MB TIF) [file pone.0005966.s003.tif]

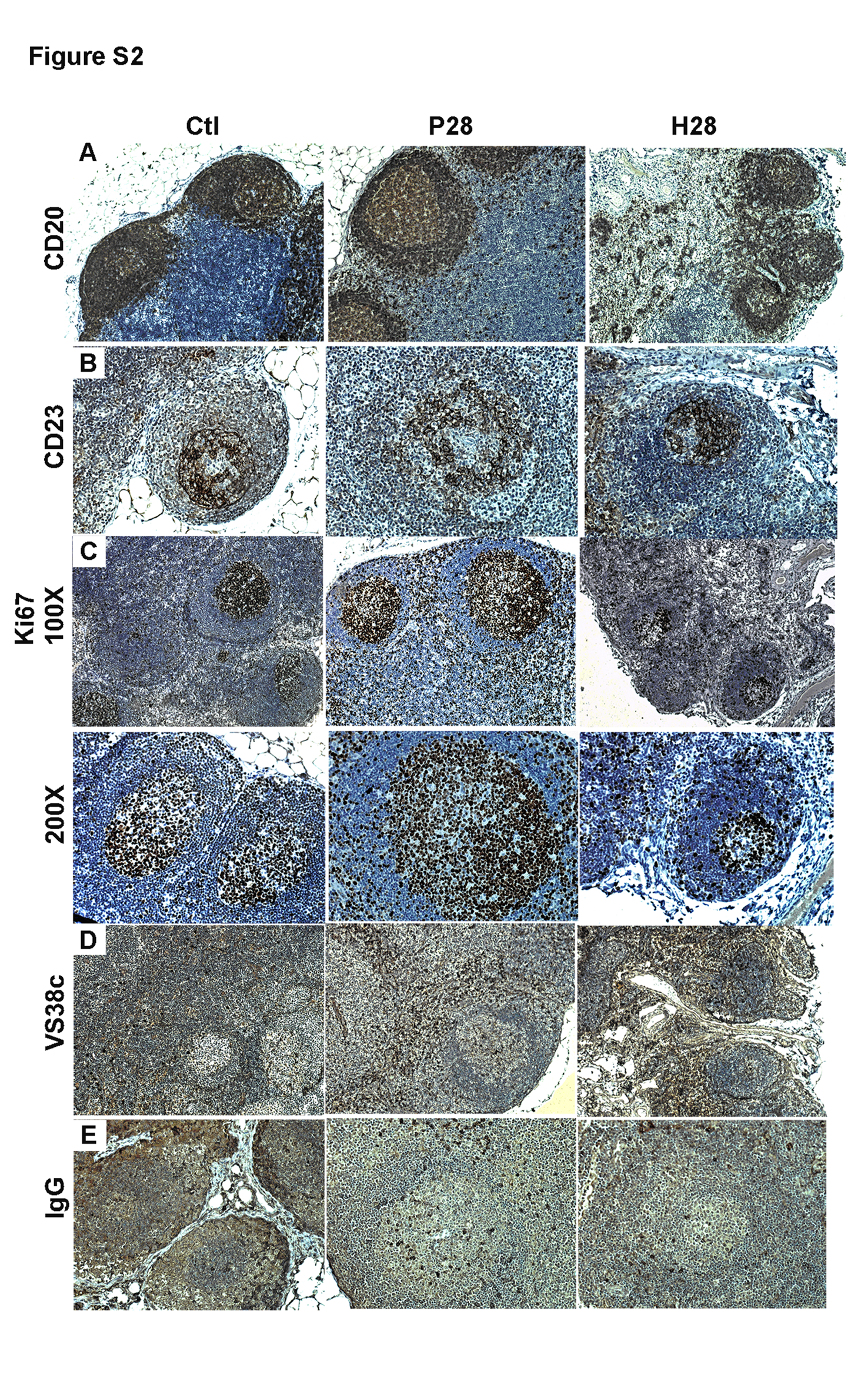

Supplement: Figure S2 — Phenotypic change within MLN B-cell areas from placebo- and HAART-treated SIV-infected animals. MLN sections from two non-infected (Ctl), P28 and H28 animals were stained for CD20 (A), CD23 (B), Ki67 (C), Vs38c (D) and IgG (E) expression. Staining from one representative animal of each group is shown. Brown indicates positive staining; cell nuclei were counterstained in blue by hematoxylin. Original magnification ×100 for A to E and ×200 for C. (8.77 MB TIF) [file pone.0005966.s004.tif]

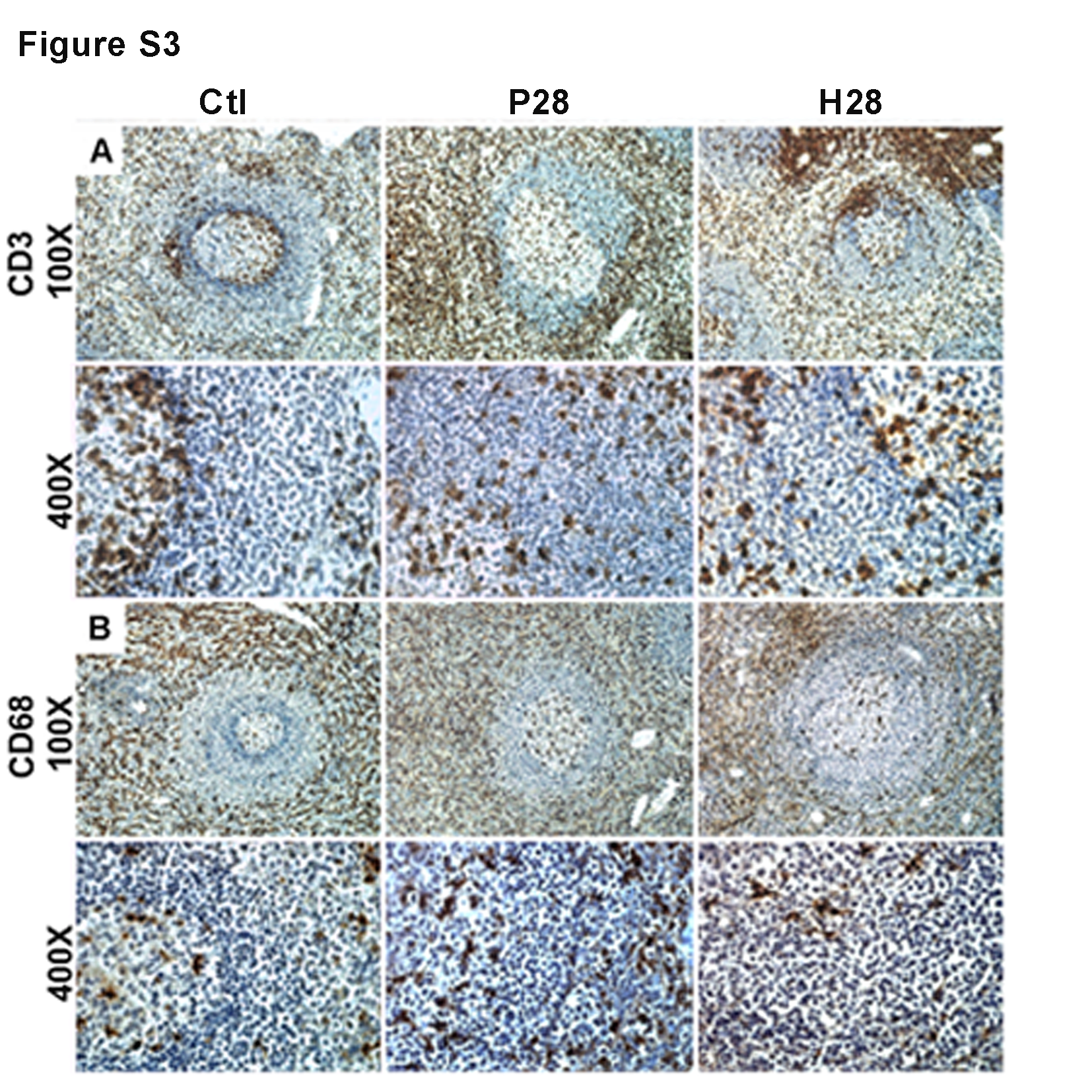

Supplement: Figure S3 — Phenotype change within mantle zone of follicles from placebo and HAART-treated SIV-infected animals. Spleen sections from two non-infected (Ctl), P28 and H28 animals were stained for CD3 (A) and CD68 (B) expression. Staining from one representative animal of each group is shown. Brown indicates positive staining; cell nuclei were counterstained in blue by hematoxylin. Original magnification 100× and 400× for both markers. (4.49 MB TIF) [file pone.0005966.s005.tif]
